# Severe acute respiratory syndrome coronavirus 2 virus-like particles induce dendritic cell maturation and modulate T cell immunity

**DOI:** 10.3389/fcimb.2022.986350

**Published:** 2022-11-09

**Authors:** Youjun Mi, Lijuan Liang, Kun Xu, Qing Li, Wenting Wang, Wenrui Dang, Jiahui Deng, Yucheng Zhi, Xuefeng Li, Jiying Tan

**Affiliations:** ^1^ Gansu Provincial Key Laboratory of Evidence Based Medicine and Clinical Translation and Lanzhou Center for Tuberculosis Research, School of Basic Medical Sciences, Lanzhou University, Lanzhou, China; ^2^ Institute of pathophysiology, School of Basic Medical Sciences, Lanzhou University, Lanzhou, China; ^3^ The First Clinical Medical College, Gansu University of Chinese Medicine, Lanzhou, China; ^4^ Institute of Immunology, School of Basic Medicine Sciences, Lanzhou University, Lanzhou, China; ^5^ Institute of Combined Western and Chinese Traditional Medicine, School of Basic Medicine Sciences, Lanzhou University, Lanzhou, China

**Keywords:** SARS-CoV-2, COVID-19, virus-like particles (VLPs), dendritic cells, T cell

## Abstract

Dendritic cells (DCs) are professional antigen-presenting cells that play an important role in both innate and acquired immune responses against pathogens. However, the role of DCs in coronavirus disease 2019 (COVID-19) is unclear. Virus-like particles (VLPs) that structurally mimic the original virus are one of the candidates COVID-19 vaccines. In the present study, severe acute respiratory syndrome coronavirus 2 (SARS-CoV-2) VLPs were used as an alternative to live virus to evaluate the interaction of the virus with DCs. The results revealed that SARS-CoV-2 VLPs induced DC maturation by augmenting cell surface molecule expression (CD80, CD86, and major histocompatibility complex class II (MHC-II)) and inflammatory cytokine production (tumor necrosis factor-α, interleukin (IL)-1β, IL-6, and IL-12p70) in DCs *via* the mitogen-activated protein kinase and nuclear factor-*κ*B signaling pathways. In addition, mature DCs induced by SARS-CoV-2 VLPs promoted T cell proliferation, which was dependent on VLPs concentration. Our results suggest that SARS-CoV-2 VLPs regulate the immune response by interacting with DCs. These findings will improve the understanding of SARS-CoV-2 pathogenesis and SARS-CoV-2 vaccine development.

## Introduction

Dendritic cells (DCs), which are professional antigen-presenting cells, play a vital role in both innate and acquired immune responses against pathogens (Stockwin et al., 2000). According to their phenotypic and functional characteristics, DCs are classified into immature and mature DCs (Sousa, 2006). Immature DCs capture and internalize pathogens *via* pattern recognition receptors (Banchereau et al., 2000), and after interaction with infectious agents, immature DCs mature. Mature DCs lose endocytic/phagocytic receptors and express higher levels of surface molecules such as CD80, CD86, and major histocompatibility complex class II (MHC-II) as well as several cytokines, including tumor necrosis factor (TNF)-α, interleukin (IL)-1β, IL-12, and IL-6 (Lee et al., 2014). Moreover, these mature DCs transport antigens to the lymph nodes for activating naive T cells (Sousa, 2022).

Preliminary studies suggest that DCs play an essential role in human coronavirus outbreaks. Yoshikawa et al. reported that severe acute respiratory syndrome coronavirus (SARS-CoV) induced DCs to express CD40 and CD86 and secrete cytokines (Yilla et al., 2005; Yoshikawa et al., 2009). One study revealed that Middle East respiratory syndrome coronavirus (MERS-CoV) could infect DCs, upregulate the expression of surface molecules on infected DCs, and induce higher expression of cytokines and chemokines in DCs infected by MERS-CoV than by SARS-CoV (Chu et al., 2014). SARS-CoV-2 invades cells *via* angiotensin-converting enzyme 2 and leads to a cytokine storm, leading to disease progression (Hoffmann et al., 2020). DCs, macrophages, neutrophils, and other immune cells are involved in the pathogenesis of coronavirus disease 2019 (COVID-19) (Mangalmurti and Hunter, 2020). Xiong et al. analyzed the transcriptomic characteristics of bronchoalveolar lavage fluid from patients with COVID-19 and found a relative increase in the levels of mature DCs (Xiong et al., 2020). The association of COVID-19 severity with the pulmonary redistribution of CD1c^+^ DCs has been reported (Sánchez-Cerrillo et al., 2020). These studies suggest that DCs participate in the lung response against SARS-CoV-2 infection. At present, the COVID-19 pandemic is still active worldwide, and vaccine and therapeutic development has been the major focus of research. However, the role of DCs in COVID-19 has not been elucidated.

Virus-like particles (VLPs) are nanoscale structures formed by viral structural proteins. VLPs lack genetic material and thus have no infectivity or pathogenicity (Mohsen et al., 2020). Therefore, they can be used as drugs, vaccines, and imaging agents as well as in drug and gene delivery (Roldão et al., 2010; Ma et al., 2012; Chung et al., 2020). VLPs have been shown to be strong DC activators by inducing DC maturation and promoting immune mediator priming based on B and T cells (Zepeda-Cervantes et al., 2020).

Research on live SARS-CoV-2 is restricted to biosafety level (BSL)-3 laboratories. Some highly pathogenic pathogens, including SARS-CoV, MERS-CoV, and Ebola virus, that require BSL-3 or -4 facilities for their handling have adopted VLPs as an alternative model for viruses (Mortola and Roy, 2004; Swenson et al., 2004; Siu et al., 2008; Wang et al., 2017). Plescia et al. established a model of SARS-CoV-2 viral budding and entry using BSL-2-level VLPs (Plescia et al., 2020). The present authors previously produced SARS-CoV-2 VLPs in insect cells using the *Bac-to-Bac* system (Mi et al., 2021). The VLPs comprised three structural proteins, including the spike (S) protein, the envelope (E) protein, and the membrane (M) protein, of SARS-CoV-2. These VLPs can serve as potential vaccine candidates as well as tools to study SARS-CoV-2 pathogenesis. Herein, SARS-CoV-2 VLPs were used as an alternative to live virus study the immune responses induced in DCs. The phenotypic and functional changes of immature DCs induced by SARS-CoV-2 VLPs were investigated.

## Methods

### Preparation of SARS-CoV-2 VLPs

The preparation and identification of SARS-CoV-2 VLPs are described previously (Mi et al., 2021). In brief, the codon-optimized *S*, *E*, and *M* genes of SARS-CoV-2 (GenBank accession no. MN908947.3) were cloned into the pFastBac triple expression vector. Recombinant baculovirus was produced using the *Bac-to-Bac* system (ThermoFisher Scientific, USA). VLPs were then obtained by infecting ExpiSf9™ insect cells with the recombinant baculovirus and purified *via* density gradient centrifugation.

SARS-CoV-2 VLPs were adsorbed onto a 400-mesh carbon-coated film for 2 min, which was then stained with 1% phosphotungstic acid for 60 s. After staining, VLP morphology was visualized using FEI Talos F200C transmission electron microscope (FEI, Czech Republic). For immunoelectron microscopy, VLPs were captured on carbon-coated copper grids. The grids were incubated with rabbit anti-spike polyclonal antibody (1:50 dilution; SinoBiological, China) at room temperature for 1 h, followed by treatment with goat anti-rabbit immunoglobulin G (1:20 dilution) (whole)-gold conjugate (10 nm) (BOSTER, China). Finally, the negative staining of the grids was performed using 1% phosphotungstic acid. VLPs were observed on the transmission electron microscope (FEI, Czech Republic) at 200 kV and 100–200 kfold magnification.

### Production and culture of DCs

DCs were produced using a modified Inaba et al. method (Inaba et al., 1992). In brief, bone marrow cells were collected from the femurs of C57BL/6 mice and lysed using red blood cell lysing buffer (ammonium chloride, 4.15 g/500 mL and 0.01 M Tris-HCl buffer; pH 7.5). The obtained cells were plated in six-well culture plates (1 × 10^6^ cells/mL; 4 mL per well) in Roswell Park Memorial Institute (RPMI) 1640 medium supplemented with 10% heat-inactivated fetal bovine serum, 100 U/mL penicillin, 100 mg/mL streptomycin, 20 ng/mL recombinant mouse (rm) granulocyte-macrophage colony-stimulating factor, and 10 ng/mL rmIL-4 at 37°C in 5% CO_2_. At days 3 and 5, half of the medium was replaced with fresh complete RPMI media with cytokines. On day 6, nonadherent cells and loosely adherent cells, which were considered immature DCs (>85% pure), were harvested for analysis or stimulation. All animal experiments were performed under the guidelines of the Council on Animal Care and Use, and the experimental protocols were reviewed and approved by the Institutional Animal Care and Use Committee of Lanzhou University.

### Cytotoxicity analysis

To investigate the cytotoxic effect of VLPs on DCs, the isolated DCs were cocultured with 5, 10, or 15 μg/mL VLPs in 12-well plates (5 × 10^5^ cells/mL). After 24, 48, or 72 h of treatment, the harvested DCs were washed with phosphate-buffered saline (PBS) and stained using fluorescein isothiocyanate (FITC)–Annexin V and propidium iodide (Sangon Biotech, China). DC cytotoxicity was then analyzed *via* flow cytometry (NovoCyte Flow Cytometer, Agilent Technologies Inc., USA).

### Measurement of DC proliferation

DCs (5 × 10^5^ cells/mL) were incubated with 5, 10, or 15 μg/mL SARS-CoV-2 VLPs at 100 μL/well in 96-well plates for 24 h. DC proliferation was evaluated using Cell Counting Kit (CCK)-8 assay (BOSTER, China). Optical density at 450 nm (OD450) was measured on a microplate reader. DC proliferation was expressed as the stimulation index (SI). SI was calculated using the following formula: SI = (ODsample well − ODblank well)/(ODnegative well − ODblank well).

### Flow cytometric analysis of surface molecule expression

On day 6, DCs were harvested, washed with cold PBS, and supplemented with 0.1% sodium azide. The cells were incubated with 0.5% bovine serum albumin in PBS for 30 min and washed with PBS. They were then stained with FITC-conjugated anti-CD11c, phycoerythrin (PE)-conjugated anti-CD80, anti-CD86, and anti-MHC-II antibodies (ThermoFisher Scientific, USA) at 4°C for 30 min. Cells were washed three times with PBS and resuspended in 500 μL PBS. Fluorescence was measured using flow cytometry, and data were analyzed using Novoexpress software.

### Antigen uptake ability of DCs *via* SARS-CoV-2 VLPs

To evaluate the antigen uptake ability of DCs, 5 × 10^5^ DCs were equilibrated at 37°C or 4°C for 45 min and then pulsed with FITC-conjugated dextran (MW 40,000, Ruixi biological technology, China) at a concentration of 1 mg/mL for 1 h; the reaction was stopped using cold staining buffer. The antigen uptake ability was measured using flow cytometry.

### Cytokine assay

TNF-α mouse uncoated enzyme-linked immunosorbent assay (ELISA) kit, IL-1β mouse uncoated ELISA kit, IL-10 mouse uncoated ELISA kit, IL-12 p70 mouse uncoated ELISA kit, interferon (IFN)-γ mouse uncoated ELISA kit, and IL-4 mouse uncoated ELISA kit were purchased from Invitrogen (ThermoFisher Scientific, USA). DCs (5 × 10^5^ cells/mL) were incubated with 5, 10, or 15 μg/mL SARS-CoV-2 VLPs for 24 h; 50 ng/mL lipopolysaccharide-treated DCs were used as the positive control. The culture supernatants were collected and evaluated for the levels of TNF-α, IL-1β, IL-10, IL-12p70, IFN-γ, and IL-4 using the respective ELISA kit according to the manufacturer’s instructions. The levels of cytokines released into culture medium were determined by measuring OD450 using a microplate reader.

### Treatment of DCs with pharmacological inhibitors of signaling pathways

All pharmacological inhibitors (Beyotime Biotechnology, China) were reconstituted in dimethyl sulfoxide (DMSO, Solarbio, China) and used at the following concentrations: U0126 (10 μM), SB203580 (20 μM), and Bay11-7082 (20 μM). DMSO (0.1%) was used as the vehicle control. In experiments with inhibitors, DCs were treated with a given inhibitor for 1 h before treatment with VLPs.

### Mixed leukocyte reaction

Syngeneic splenocytes isolated from C57BL/6 mice were lysed using red blood cell lysing buffer (BOSTER, China). After washing twice with PBS, the cells were resuspended in complete RPMI 1640 (Invitrogen, China) and subsequently used for MLR. To test their allogeneic stimulatory activity, DCs were treated with SARS-CoV-2 VLPs for 48 h and then cocultured with the obtained splenic cells at a ratio of 1:20, 1:40, or 1:80 in 96-well plates for 72 h, followed by incubation in 10 μL CCK-8 solution (BOSTER, China) at 37°C for 4 h. OD450 was measured using a microplate reader. SI was calculated using the following formula: SI = (ODsample well − ODblank well)/(ODnegative well − ODblank well).

To further investigate the proliferation of CD4^+^ and CD8^+^ T cells in splenocytes, the splenic cells were resuspended in 5 μM carboxyfluorescein diacetate succinimidyl ester (CFSE, Beyotime Biotechnology, China) in DMSO for 10 min at room temperature. DCs (5 × 10^4^ cells/well), treated with 10 μg/mL VLPs for 48 h, were cocultured with the CFSE-labeled autologous naive T cells (1 × 10^6^ cells) derived from spleens at a ratio of 1:20 in 12-well plates for 72 h, and the culture supernatants were collected for cytokine analysis. The cocultured cells were collected and stained with PE-conjugated anti-CD4 and PerCP-conjugated anti-CD8 antibodies (ThermoFisher Scientific, USA). Then, CD4^+^ and CD8^+^ T cell proliferation was assessed *via* the flow cytometric analysis of CFSE dilution of T cells.

### Statistical analyses

All experiments were repeated three times. The levels of significance for comparison between the samples were determined *via* Bonferroni’s multiple comparison test distribution using a statistical software (GraphPad Prism 9.0, San Diego, CA, USA). The data in the graphs are expressed as mean ± standard error. *P < 0.05 or **P < 0.01 denotes statistical significance.

## Results

### Preparation of SARS-CoV-2 VLPs

**Figure 1:**
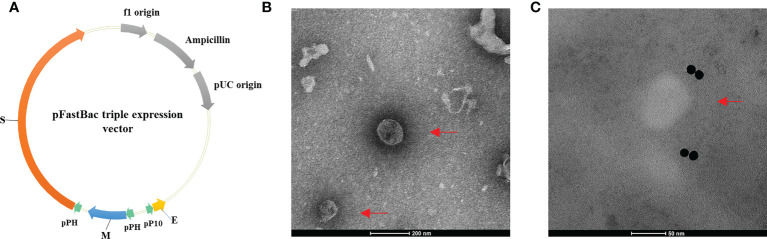
Construction of the pFastBac triple expression vector and the morphology of SARS-CoV-2 VLPs. **(A)** Construction of the pFastBac triple expression vector. The codon-optimized *S*, *E*, and *M* genes of SARS-CoV-2 (GenBank accession No. MN908947.3) were inserted into the pFastBac dual vector to construct a new pFastBac triple expression vector. **(B, C)** Morphology of SARS-CoV-2 VLPs as shown in transmission electron microscope. Red arrows denote VLPs. **(B)** SARS-CoV-2 VLPs stained with phosphotungstic acid. Scale bar = 200 nm. **(C)** Negative stain electron microscopy reveals SARS-CoV-2 VLPs specifically labeled with immunogold. Scale bar = 50 nm.

SARS-CoV-2 VLPs were prepared according to our previous study [23]. For the expression of the E, M, and S proteins of SARS-CoV-2, the codon-optimized *S*, *E*, and *M* genes of SARS-CoV-2 (GenBank accession no. MN908947.3) were inserted into the pFastBac expression vector (**Figure 1A**). SARS-CoV-2 VLPs were then produced in insect cells using the *Bac-to-Bac* system. Using transmission electron microscopy, VLP morphology was observed. As shown in **Figures 1B, C**, VLP appeared as spherical particles with a diameter of approximately 100 nm, similar to the native SARS-CoV-2.

### Cytotoxicity of SARS-CoV-2 VLPs

**Figure 2:**
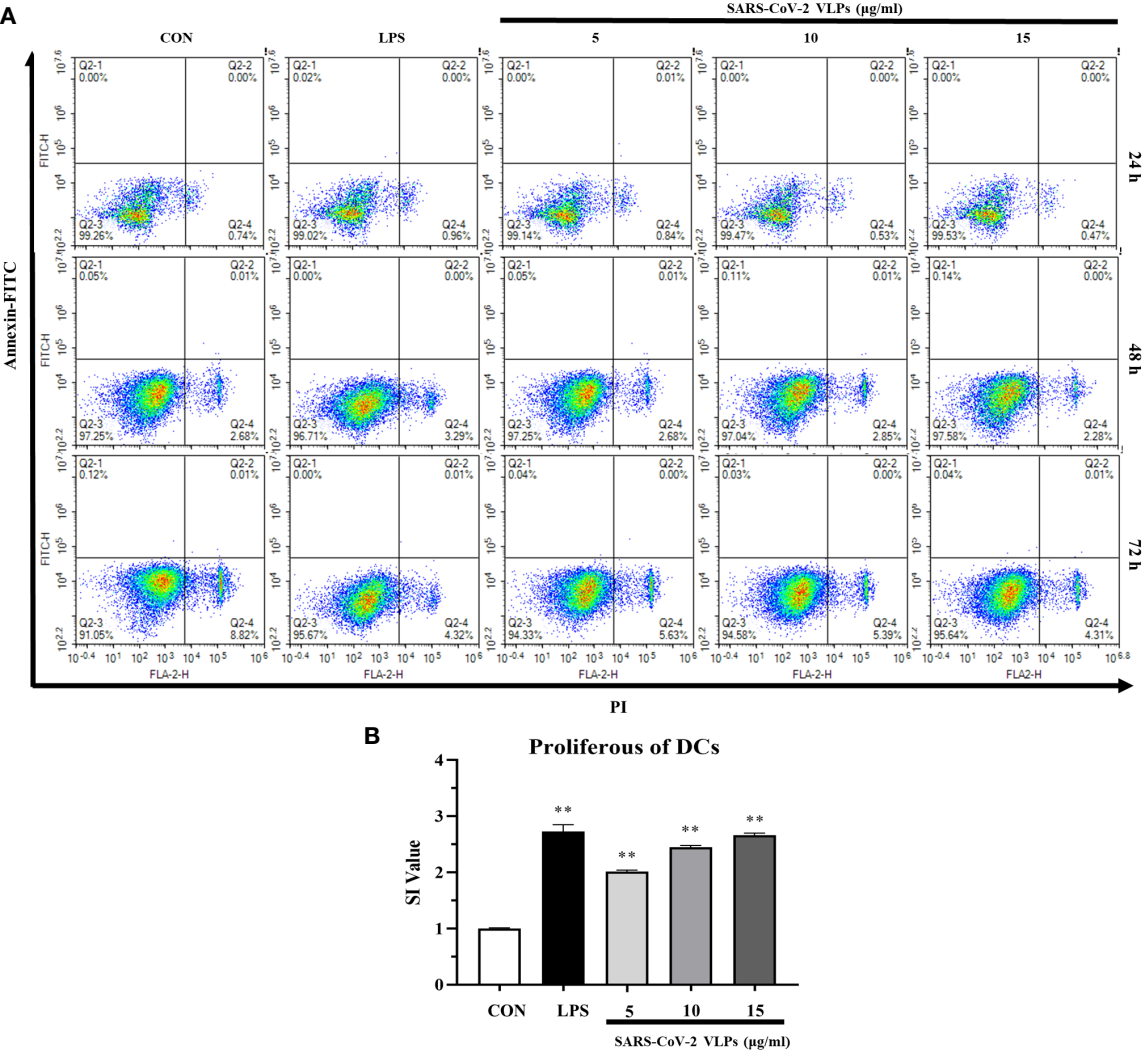
SARS-CoV-2 VLPs are not cytotoxic to dendritic cells (DCs) and stimulate DCs proliferation. **(A)** DCs were cultured with 5, 10, or 15 μg/mL SARS-CoV-2 VLPs for 24, 48, or 72 h. Then, the treated cells were stained with FITC–Annexin V and propidium iodide and analyzed *via* flow cytometry; untreated DCs are control (CON), and the percentage of cells is shown in each panel. **(B)** DCs were cultured with 5, 10, or 15 μg/mL SARS-CoV-2 VLPs for 24 h, and the proliferation of DCs was evaluated using the CCK-8 kit. The stimulation index (SI) value was applied to indicate the proliferation level of DCs. All data are expressed as means ± SE; **P < 0.01 versus untreated control (CON).

To investigate the cytotoxicity of SARS-CoV-2 VLPs against DCs, DCs were collected and incubated with SARS-CoV-2 VLPs at different time points. Cell viability was assessed using Annexin V and propidium iodide staining at different time points. As shown in **Figure 2A**, compared with the control, VLPs of up to 15 μg/mL concentration displayed no cellular toxicity against DCs. Moreover, the particles could induce DC proliferation (**Figure 2B**).

### SARS-CoV-2 VLPs induce DC maturation

**Figure 3:**
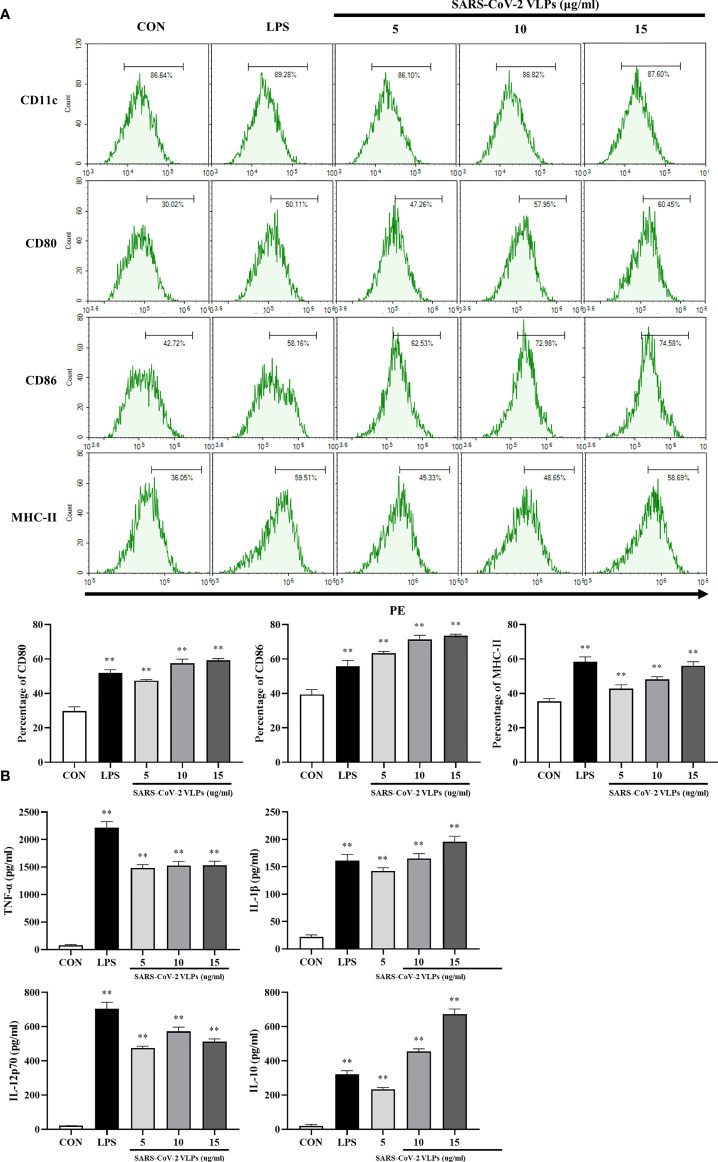
SARS-CoV-2 VLPs induce DCs maturation. DCs were treated with SARS-CoV-2 VLPs (5, 10, or 15 μg/mL) or LPS (50 ng/mL) for 24 h. Untreated DCs were used as control (CON). **(A)** Flow cytometry was used to analyze the expression of surface molecule markers on DCs. The percentages are indicated in the histogram. Results are the representative of three experiments with similar data. **(B)** ELISA was used to determine the levels of TNF-α, IL-1β, IL-12p70, and IL-10 in SARS-CoV-2 VLP-treated or LPS-treated DCs. Data are the means ± SE of three experiments. **P < 0.01 versus untreated control (CON).

To investigate the effect of SARS-CoV-2 VLPs on the maturation of DCs, the phenotypic alteration and cytokine secretion of DCs were evaluated. The results showed that VLP-treated DCs had increased levels of costimulatory molecules (such as CD80 and CD86) and cell surface markers (such as MHC class II) (**Figure 3A**). As various cytokines secreted by DCs have effects on DC maturation and T cell polarization, the levels of several immunomodulatory cytokines secreted by DCs, including TNF-α, IL-1β, IL-12p70, and IL-10, were investigated. The results revealed that SARS-CoV-2 VLPs induced DCs to secrete TNF-α, IL-1β, IL-10, and IL-12p70 (**Figure 3B**).

**Figure 4:**
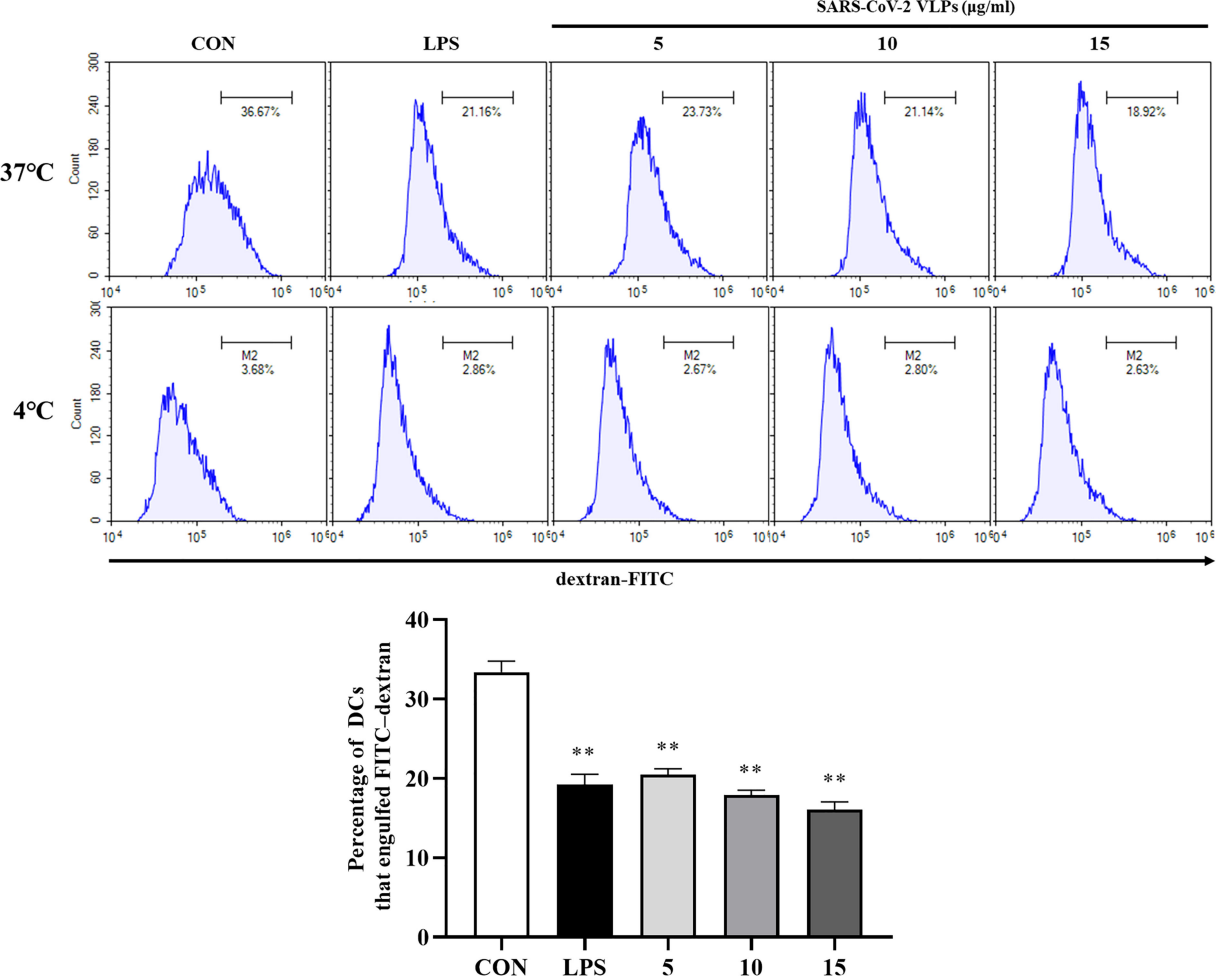
SARS-CoV-2 VLP-treated DCs had diminished endocytic capacity. The endocytic capacity of DCs was assessed using FITC-conjugated dextran uptake system at 37°C and 4°C (as a negative control) *via* flow cytometry. Values are the means ± SE of three independent experiments. **P < 0.01 versus untreated DCs (CON).

In many cases, pathogen recognition and uptake are accompanied by the maturation of DCs; the endocytic capacity of DCs is downregulated during their maturation, and immature DCs have a higher antigen endocytic capacity than mature DCs (Guermonprez et al., 2002). Therefore, the influence of SARS-CoV-2 VLPs on the endocytic capacity of DC was evaluated using the dextran–FITC uptake experiment. Dextran–FITC is mainly absorbed by the mannose receptor, a commonly used model substrate for studying pinocytosis and phagocytosis (Sallusto et al., 1995; Monti et al., 2003). DCs incubated with dextran–FITC at 37°C accumulated this marker in a time-dependent manner and can be detected by flow cytometry (Sallusto et al., 1995). The flow cytometry analysis revealed that SARS-CoV-2 VLP-treated DCs had reduced endocytic capacity, which was expected for mature DCs (**Figure 4**).

In the present study, after the interaction between DCs and SARS-CoV-2 VLPs, DCs downregulated the antigen uptake capacity, increased surface molecule expression, and secreted cytokines. These results suggest that immature DCs become mature after undergoing phenotypic and functional changes and that SARS-CoV-2 VLPs are potent inducers of DC maturation.

### SARS-CoV-2 VLPs activate DCs *via* the mitogen-activated protein kinase and nuclear factor- *κ*B signaling pathways

**Figure 5:**
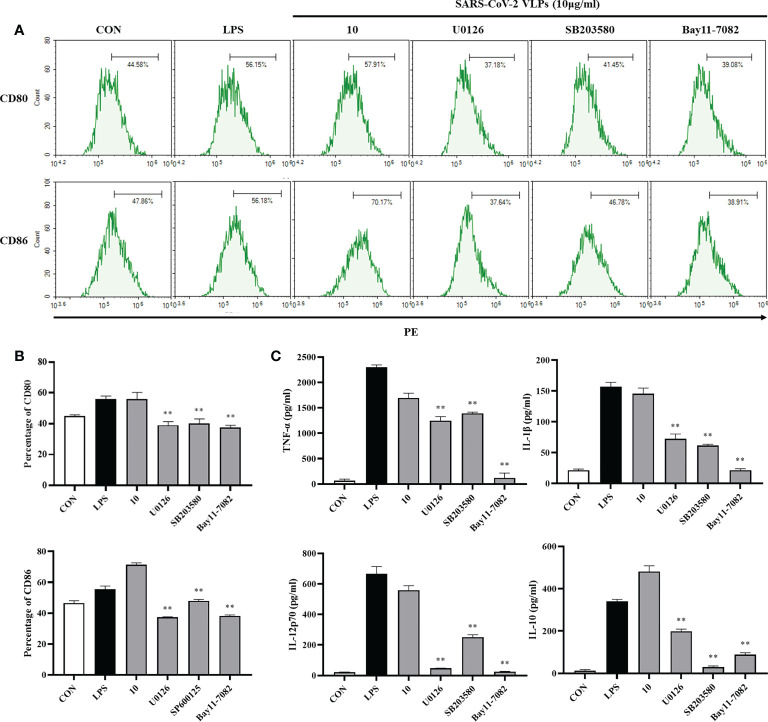
Involvement of the MAPK signaling pathways in SARS-CoV-2 VLP-induced DC maturation. DCs were treated with the pharmacological inhibitors of ERK1/2 (U0126, 10 M), p38 (SB203580, 20M), NF-*κ*B (Bay11-7082, 20 M), or DMSO (control) for 1 h prior to stimulation with 10 μg/mL SARS-CoV-2 VLPs for 24 h **(A, B)** DCs were stained with anti-CD80 and anti-CD86 antibodies, and the expression of CD80 and CD86 was analyzed using flow cytometry. **(C)** Culture supernatant concentration of TNF-α, IL-1β, IL-12p70, and IL-10 were measured *via* ELISA. Values are the means ± SE of three independent experiments. **P < 0.01 versus SARS-CoV-2 VLPs-treated groups (10 μg/mL).

NF-*κ*B and MAPKs play a crucial role in DC maturation (Rescigno et al., 1998). Whether SARS-CoV-2 VLPs activate MAPKs and NF-κB in DCs was then investigated. U0126 permeabilizes cells and specifically inhibits the ability of mitogen-activated protein kinase/ERK kinase (MEK) to phosphorylate extracellular signal-regulated kinase (ERK) without affecting the p38 and Jun NH_2_-terminal kinase (JNK) pathways (DeSilva et al., 1998). SB 203580 is a widely used specific inhibitor of p38 MAPK that subsequently inhibits the activation of mitogen activated protein kinase-activated protein kinase (MAPKAP kinase)-2 and MAPKAP kinase-3 (Hashimoto et al., 1999). BAY 11-7082, a known NF-*κ*B inhibitor, antagonizes I*κ*B kinase β and prevents the nuclear translocation of NF-*κ*B (Kim et al., 2015; Irrera et al., 2017). In the present study, DCs were pretreated with the ERK1/2 inhibitor U0126, the p38 inhibitor SB203580, and the NF-*κ*B inhibitor Bay11-7082 and subsequently stimulated with SARS-CoV-2 VLPs. DC maturation was evaluated by measuring surface markers and inflammatory cytokines, and the results revealed that U0126, SB203580, and Bay11-7082 significantly downregulated the SARS-CoV-2 VLP-induced expression of the DC maturation markers CD80 and CD86 (**Figure 5A**). In addition, the VLP-induced expression of TNF-α, IL-1β, IL-12p70, and IL-10 by DCs was markedly reduced after pretreatment with the pharmacological inhibitors (**Figure 5B**). These results indicated that SARS-CoV-2 VLPs activated the MAPK and NF-*κ*B signaling pathways and that these signaling pathways play a crucial role in DC maturation.

### SARS-CoV-2 VLPs promote naive T cell proliferation

**Figure 6:**
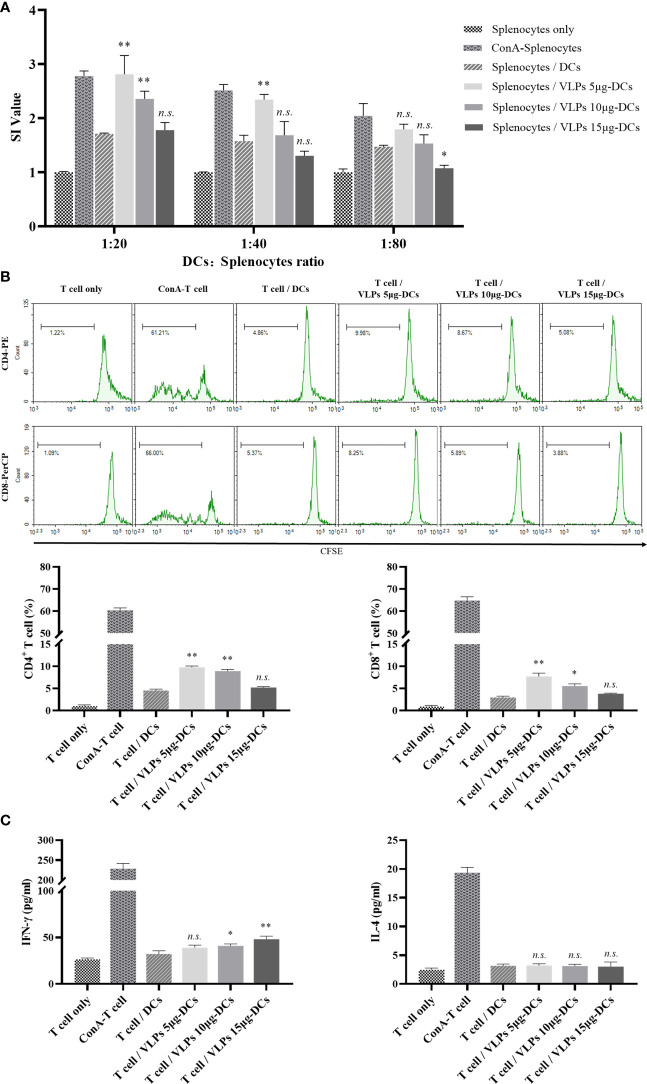
SARS-CoV-2 VLPs induce DCs to activate T cells ex vivo. **(A)** DCs were cultured with SARS-CoV-2 VLPs for 48 h and then cocultured with splenocytes at a ratio of 1:20, 1:40, and 1:80 for 72 h Splenocytes alone and splenocytes cocultured with untreated DCs served as controls, whereas concanavalin A (ConA)-treated splenocytes served as the positive control. The proliferation of splenocytes was measured using the CCK-8 kit. All data are the representative of three independent assays and expressed as mean ± SE; *P < 0.05 or ** <0.01 versus splenocytes cocultured with untreated DCs. *n.s.* not statistically significant. **(B)** Splenocytes were prestained with 5 μM CFSE and cocultured with SARS-CoV-2 VLP-treated DCs at a ratio of 20:1 for 72 h The proliferation of CD4^+^and CD8^+^ T cells in spleen cells were assessed *via* flow cytometry. All data are the representative of three independent assays and expressed as mean ± SE; *P < 0.05 or ** <0.01 versus T cells cocultured with untreated DCs. *n.s.* not statistically significant. **(C)** Culture supernatants obtained from experiments in B were harvested, and IFN-γ and IL-4 were measured using ELISA. All data are the representative of three independent assays and expressed as mean ± SE; *P < 0.05 or ** <0.01 versus T cells cocultured with untreated DCs. *n.s.* not statistically significant.

Mature DCs have the ability to activate antigen-specific naive T cells to initiate adaptive immune responses (Stockwin et al., 2000). To investigate whether SARS-CoV-2 VLPs can induce DC maturation and promote naive T cell proliferation, MLR assay was performed. The splenocytes of C57BL/6 mice were cocultured with SARS-CoV-2 VLP-treated DCs, and the results showed that SARS-CoV-2 VLP-treated DCs enhanced the proliferation of splenocytes. Of note, cell proliferation was negatively correlated with VLP concentration (**Figure 6A**). CFSE can be used to monitor the number of T cell divisions during proliferation, which was assessed using flow cytometry (Hawkins et al., 2007). DCs treated with SARS-CoV-2 VLPs at low concentrations, but not at high concentrations, promoted the proliferation of CD8^+^ and CD4^+^ T cells (**Figure 6B**). These results suggest that VLPs provide a potent stimulus for the proliferation of CD8^+^ and CD4^+^ T cells *via* DC activation. Cytokine production was also evaluated under MLR conditions, which showed that a higher level of IFN-γ was produced by T cells primed with SARS-CoV-2 VLP-treated DCs (**Figure 6C**). This result indicated that mature DCs induced by SARS-CoV-2 VLPs drive T cell polarization toward the Th1 phenotype.

## Discussion

SARS-CoV-2 is the causative agent of COVID-19 (Zhu et al., 2020). However, few functional interactions have been identified between the host cells and SARS-CoV-2. DCs are professional antigen-presenting cells with a unique ability to initiate and regulate cell-mediated immune responses. While the key importance of DCs in coronavirus diseases has been reported (Marzi et al., 2004; Cervantes-Barragan et al., 2007; Yoshikawa et al., 2009; Law et al., 2009; Chu et al., 2014; Chang et al., 2022), its role in the pathogenesis of COVID-19 has not been sufficiently studied. In the present study, the functional roles mediated by SARS-CoV-2 VLPs in their interaction with DCs were evaluated.

Studies have shown that exogenous signaling induces DCs to downregulate antigen uptake and upregulate maturation markers (De Smedt et al., 1996; Banchereau et al., 2000; Manickasingham and Reis e Sousa, 2000). The upregulation of certain molecules, including MHC, CD40, CD80, CD83, and CD86, correlates with T cell-priming ability (Banchereau et al., 2000). The exposure of DCs to the infectious SARS-CoV leads to the phenotypic maturation of DCs, with the increased expression of MHC-II and costimulatory molecules (Tseng et al., 2005). SARS-CoV VLPs have also been shown to induce higher levels of CD83, CD40, CD86, and CD80 expression on DCs (Bai et al., 2008). In line with these studies, the present study results demonstrated that SARS-CoV-2 VLPs induce DC to downregulate dextran–FITC uptake and upregulate CD80, CD86, and MHC-II surface markers (**Figures 4**, **3A**).

DC maturation results in proinflammatory cytokine production that can subsequently activate innate and adaptive immune responses as well as mediate immunopathology (Liu, 2022). MERS-CoV-infected monocyte-derived DCs highly expressed IFN, IFN-inducible protein 10 (IP-10), IL-12, and RANTES (Chu et al., 2014). After abortive infection with SARS-CoV, DCs upregulated the expression of chemokines IP-10, monocyte chemoattractant protein 1, macrophage inflammatory protein 1α, and RANTES (Chen and Subbarao, 2007). Incubating DCs with SARS-CoV VLPs significantly enhanced the expression levels of IL-6, IL-10, and TNF-α (Liu, 2022). The present study showed that SARS-CoV-2 VLP-induced DCs increased the production of TNF-α, IL-1β, IL-12p70, and IL-10 (**Figure 3B**).

DC maturation is accompanied by downregulated antigen uptake capacity, increased surface molecule expression, and increased cytokines secretion (De Smedt et al., 1996; Banchereau et al., 2000; Manickasingham and Reis e Sousa, 2000; Guermonprez et al., 2002; Liu, 2022). The present study results indicated that SARS-CoV-2 VLPs can interact with DCs to induce DC maturation and activation.

It has been reported that the MAPK and NF-*κ*B signaling pathways are involved in DC maturation (Richards et al., 2002; Byun et al., 2012). In line with these studies, SARS-CoV-2 VLPs significantly increased the expression of surface molecules and proinflammatory cytokine secretion by activating the MAPK and NF-*κ*B signaling pathways (**Figure 5**). Thus, the study findings indicated that these two signaling pathways are essential for the SARS-CoV-2 VLP-induced maturation of DCs.

Mature DCs induce naive T cell proliferation and differentiate into effector T cells in the lymph nodes (Banchereau and Steinman, 1998). DCs regulate the T cell differentiation process *via* antigen presentation and by triggering specialized cytokine microenvironments. The differentiation of naive T cells into appropriate T cell subtypes is critical for antiviral immunity. CD4^+^ and CD8^+^ T cells are important immune components against intracellular viral infection. CD4^+^ T cells enhance the antibody and cytotoxic T lymphocyte responses. CD8^+^ T cells recognize virus-infected cells and eliminate them to limit viral spread (Young, 2022). To precisely characterize the SARS-CoV-2 VLP activity on DC and T cell interactions, the syngeneic MLR assay was performed, which revealed that SARS-CoV-2 VLP treatment enhanced CD4^+^ and CD8^+^ T cell proliferation (**Figures 6A, B**). In addition, T cells cocultured with SARS-CoV-2 VLP-treated DCs increased the expression of IFN-γ but not of IL-4 (**Figure 6C**). T cell proliferation and IFN-γ production indicated that the SARS-CoV-2 VLP-induced maturation of DCs was responsible for presenting the antigens to T cells and influencing the subsequent adaptive immune responses.

In COVID-19, high T cell activity response is associated with milder disease, whereas a poor T cell response is associated with severe disease (Young, 2022). In the present study, interestingly, the proliferation of both CD4^+^ and CD8^+^ T cells was negatively correlated with the concentration of VLPs. Thus, the impact of changes in the SARS-CoV-2 viral load on DC–T cell interactions may have contributed to the different outcomes of COVID-19, suggesting that the role of DCs in COVID-19 needs to be further clarified.

Taken together, these findings demonstrate that SARS-CoV-2 VLPs can activate DCs and initiate the adaptive immune response. The present study results may contribute to a better understanding of the mechanisms involved in the immune response against SARS-CoV-2 and provide knowledge for the rational design and development of future COVID-19 vaccines.

## Data availability statement

The datasets presented in this study can be found in online repositories. The names of the repository/repositories and accession number(s) can be found below: NCBI, GenBank accession no. MN908947.3.

## Ethics statement

The animal study was reviewed and approved by Institutional Animal Care and Use Committee of Lanzhou University.

## Author contributions

YM and LL contributed to conception and design of the study and wrote the first draft of the manuscript. KX, QL, WW, WD, JD, and YZ carried out the experiments. LL performed the statistical analysis. JT revised the manuscript. All authors contributed to the article and approved the submitted version.

## Funding

This research was funded by the Gansu Science and Technology Project (21JR7RA443), Science and Technology Innovation Project of Gansu Provincial Department of Education (2022B-118), Main Research and Development Program of Gansu Province in 2020 (20YF8FA072), Lanzhou Science and Technology Planning Project (2020-XG-33), Key Laboratory of Dunhuang Medical and Translational Education Ministry Project (DHXY20-08).

## Conflict of interest

The authors declare that the research was conducted in the absence of any commercial or financial relationships that could be construed as a potential conflict of interest.

## Publisher’s note

All claims expressed in this article are solely those of the authors and do not necessarily represent those of their affiliated organizations, or those of the publisher, the editors and the reviewers. Any product that may be evaluated in this article, or claim that may be made by its manufacturer, is not guaranteed or endorsed by the publisher.

## References

[B1] Bai B. Hu Q. Hu H. Zhou P. Shi Z. Meng J. . (2008). Virus-like particles of sars-like coronavirus formed by membrane proteins from different origins demonstrate stimulating activity in human dendritic cells. PloS One 3 (7), e2685. doi: 10.1371/journal.pone.0002685 18628832 PMC2441860

[B2] Banchereau J. Briere F. Caux C. Davoust J. Lebecque S. Liu Y. J. . (2000). Immunobiology of dendritic cells. Annu. Rev. Immunol. 18, 767–811. doi: 10.1146/annurev.immunol.18.1.767 10837075

[B3] Banchereau J. Steinman R. M. (1998). Dendritic cells and the control of immunity. Nature 392 (6673), 245–252. doi: 10.1038/32588 9521319

[B4] Byun E. H. Kim W. S. Kim J. S. Jung I. D. Park Y. M. Kim H. J. . (2012). Mycobacterium tuberculosis Rv0577, a novel Tlr2 agonist, induces maturation of dendritic cells and drives Th1 immune response. FASEB J. 26 (6), 2695–2711. doi: 10.1096/fj.11-199588 22415304

[B5] Cervantes-Barragan L. Züst R. Weber F. Spiegel M. Lang K. S. Akira S. . (2007). Control of coronavirus infection through plasmacytoid dendritic-Cell-Derived type I interferon. Blood 109 (3), 1131–1137. doi: 10.1182/blood-2006-05-023770 16985170 PMC8254533

[B6] Chang T. Yang J. Deng H. Chen D. Yang X. Tang Z.-H. (2022). Depletion and dysfunction of dendritic cells: Understanding sars-Cov-2 infection. Front. Immunol. 13. doi: 10.3389/fimmu.2022.843342 PMC8898834 35265087

[B7] Chen J. Subbarao K. (2007). The immunobiology of sars. Annu. Rev. Immunol. 25 (1), 443–472. doi: 10.1146/annurev.immunol.25.022106.141706 17243893

[B8] Chung Y. H. Cai H. Steinmetz N. F. (2020). Viral nanoparticles for drug delivery, imaging, immunotherapy, and theranostic applications. Adv. Drug Delivery Rev. S0169-409X (20), 30070–30073. doi: 10.1016/j.addr.2020.06.024 PMC7320870 32603813

[B9] Chu H. Zhou J. Wong B. H. Li C. Cheng Z. S. Lin X. . (2014). Productive replication of middle East respiratory syndrome coronavirus in monocyte-derived dendritic cells modulates innate immune response. Virology 454-455, 197–205. doi: 10.1016/j.virol.2014.02.018 24725946 PMC7111975

[B10] DeSilva D. R. Jones E. A. Favata M. F. Jaffee B. D. Magolda R. L. Trzaskos J. M. . (1998). Inhibition of mitogen-activated protein kinase kinase blocks T cell proliferation but does not induce or prevent anergy. J. Immunol. 160 (9), 4175–4181. 9574517

[B11] De Smedt T. Pajak B. Muraille E. Lespagnard L. Heinen E. De Baetselier P. . (1996). Regulation of dendritic cell numbers and maturation by lipopolysaccharide in vivo. J. Exp. Med. 184 (4), 1413–1424. doi: 10.1084/jem.184.4.1413 8879213 PMC2192842

[B12] Guermonprez P. Valladeau J. Zitvogel L. Théry C. Amigorena S. (2002). Antigen presentation and T cell stimulation by dendritic cells. Annu. Rev. Immunol. 20 (1), 621–667. doi: 10.1146/annurev.immunol.20.100301.064828 11861614

[B13] Hashimoto S. Matsumoto K. Gon Y. Furuichi S. Maruoka S. Takeshita I. . (1999). Thioredoxin negatively regulates P38 map kinase activation and il-6 production by tumor necrosis factor-alpha. Biochem. Biophys. Res. Commun. 258 (2), 443–447. doi: 10.1006/bbrc.1999.0658 10329406

[B14] Hawkins E. D. Hommel M. Turner M. L. Battye F. L. Markham J. F. Hodgkin P. D. (2007). Measuring lymphocyte proliferation, survival and differentiation using cfse time-series data. Nat. Protoc. 2 (9), 2057–2067. doi: 10.1038/nprot.2007.297 17853861

[B15] Hoffmann M. Kleine-Weber H. Schroeder S. Krüger N. Herrler T. Erichsen S. . (2020). Sars-Cov-2 cell entry depends on Ace2 and Tmprss2 and is blocked by a clinically proven protease inhibitor. Cell 181 (2), 271–2808. doi: 10.1016/j.cell.2020.02.052 32142651 PMC7102627

[B16] Inaba K. Inaba M. Romani N. Aya H. Deguchi M. Ikehara S. . (1992). Generation of Large numbers of dendritic cells from mouse bone marrow cultures supplemented with Granulocyte/Macrophage colony-stimulating factor. J. Exp. Med. 176 (6), 1693–1702. doi: 10.1084/jem.176.6.1693 1460426 PMC2119469

[B17] Irrera N. Vaccaro M. Bitto A. Pallio G. Pizzino G. Lentini M. . (2017). Bay 11-7082 inhibits the nf-Kb and Nlrp3 inflammasome pathways and protects against imq-induced psoriasis. Clin. Sci. (Lond) 131 (6), 487–498. doi: 10.1042/cs20160645 28096316

[B18] Kim H. R. Shin D. Y. Chung K. H. (2015). The role of nf-Kb signaling pathway in polyhexamethylene guanidine phosphate induced inflammatory response in mouse macrophage Raw264.7 cells. Toxicol. Lett. 233 (2), 148–155. doi: 10.1016/j.toxlet.2015.01.005 25578230

[B19] Law H. K. W. Cheung C. Y. Sia S. F. Chan Y. O. Peiris J. S. M. Lau Y. L. (2009). Toll-like receptors, chemokine receptors and death receptor ligands responses in sars coronavirus infected human monocyte derived dendritic cells. BMC Immunol. SCIIF 3 10, 35. doi: 10.1186/1471-2172-10-35 PMC2700820 19505311

[B20] Lee S. J. Shin S. J. Lee S. J. Lee M. H. Kang T. H. Noh K. T. . (2014). Mycobacterium abscessus Mab2560 induces maturation of dendritic cells *Via* toll-like receptor 4 and drives Th1 immune response. BMB Rep. 47 (9), 512–517. doi: 10.5483/bmbrep.2014.47.9.001 24667171 PMC4206727

[B21] Liu Y. J. (2022). Dendritic cell subsets and lineages, and their functions in innate and adaptive immunity. Cell 106 (3), 259–262. doi: 10.1016/s0092-8674(01)00456-1 11509173

[B22] Mangalmurti N. Hunter C. A. (2020). Cytokine storms: Understanding covid-19. Immunity 53 (1), 19–25. doi: 10.1016/j.immuni.2020.06.017 PMC7321048 32610079

[B23] Manickasingham S. Reis e Sousa C. (2000). Microbial and T cell-derived stimuli regulate antigen presentation by dendritic cells in vivo. J. Immunol. 165 (9), 5027–5034. doi: 10.4049/jimmunol.165.9.5027 11046031

[B24] Ma Y. Nolte R. J. M. Cornelissen J. J. L. M. (2012). Virus-based nanocarriers for drug delivery. Adv. Drug Delivery Rev. 64 (9), 811–825. doi: 10.1016/j.addr.2012.01.005 22285585

[B25] Marzi A. Gramberg T. Simmons G. Möller P. Rennekamp A. J. Krumbiegel M. . (2004). Dc-sign and dc-signr interact with the glycoprotein of marburg virus and the s protein of severe acute respiratory syndrome coronavirus. J. Virol. 78 (21), 12090–12095. doi: 10.1128/jvi.78.21.12090-12095.2004 15479853 PMC523257

[B26] Mi Y. Xie T. Zhu B. Tan J. Li X. Luo Y. . (2021). Production of sars-Cov-2 virus-like particles in insect cells. Vaccines 9 (6), 554. doi: 10.3390/vaccines9060554 34073159 PMC8227081

[B27] Mohsen M. O. Augusto G. Bachmann M. F. Mohsen M. O. Augusto G. Bachmann M. F. (2020). The 3ds in virus-like particle based-vaccines: "Design, delivery and dynamics". Immunol. Rev 296(1), 155–168. doi: 10.1111/imr.12863 PMC7496916 32472710

[B28] Monti P. Mercalli A. Leone B. E. Valerio D. C. Allavena P. Piemonti L. (2003). Rapamycin impairs antigen uptake of human dendritic cells. Transplantation 75 (1), 137–145. doi: 10.1097/00007890-200301150-00025 12544886

[B29] Mortola E. Roy P. (2004). Efficient assembly and release of sars coronavirus-like particles by a heterologous expression system. FEBS Lett. 576 (1-2), 174–178. doi: 10.1016/j.febslet.2004.09.009 15474033 PMC7126153

[B30] Plescia C. B. David E. A. Patra D. Sengupta R. Amiar S. Su Y. . (2020). Sars-Cov-2 viral budding and entry can be modeled using bsl-2 level virus-like particles. J. Biol. Chem. 296, 100103. doi: 10.1074/jbc.RA120.016148 33214224 PMC7832013

[B31] Rescigno M. Martino M. Sutherland C. L. Gold M. R. Ricciardi-Castagnoli P. (1998). Dendritic cell survival and maturation are regulated by different signaling pathways. J. Exp. Med. 188 (11), 2175–2180. doi: 10.1084/jem.188.11.2175 9841930 PMC2212396

[B32] Richards J. Le Naour F. Hanash S. Beretta L. (2002). Integrated genomic and proteomic analysis of signaling pathways in dendritic cell differentiation and maturation. Ann. NY Acad. Sci. 975, 91–100. doi: 10.1111/j.1749-6632.2002.tb05944.x 12538157

[B33] Roldão A. Mellado M. C. M. Castilho L. R. Carrondo M. J. T. Alves P. M. (2010). Virus-like particles in vaccine development. Expert Rev. Vaccines 9 (10), 1149–1176. doi: 10.1586/erv.10.115 20923267

[B34] Sallusto F. Cella M. Danieli C. Lanzavecchia A. (1995). Dendritic cells use macropinocytosis and the mannose receptor to concentrate macromolecules in the major histocompatibility complex class ii compartment: Downregulation by cytokines and bacterial products. J. Exp. Med. 182 (2), 389–400. doi: 10.1084/jem.182.2.389 7629501 PMC2192110

[B35] Sánchez-Cerrillo I. Landete P. Aldave B. Sánchez-Alonso S. Sánchez-Azofra A. Marcos-Jiménez A. . (2020). Covid-19 severity associates with pulmonary redistribution of Cd1c+ dcs and inflammatory transitional and nonclassical monocytes. J. Clin. Invest. 130 (12), 6290–6300. doi: 10.1172/jci140335 32784290 PMC7685723

[B36] Siu Y. L. Teoh K. T. Lo J. Chan C. M. Kien F. Escriou N. . (2008). E, and n structural proteins of the severe acute respiratory syndrome coronavirus are required for efficient assembly, trafficking, and release of virus-like particles. J. Virol. 82 (22), 11318–11330. doi: 10.1128/jvi.01052-08 18753196 PMC2573274

[B37] Sousa C. R. E. (2006). Dendritic cells in a mature age. Nat. Rev. Immunol. 6 (6), 476–483. doi: 10.1038/nri1845 16691244

[B38] Sousa C. R. E. (2022). Dendritic cells as sensors of infection. Immunity 14 (5), 495–498. doi: 10.1016/s1074-7613(01)00136-4 11371351

[B39] Stockwin L. H. McGonagle D. Martin I. G. Blair G. E. (2000). Dendritic cells: Immunological sentinels with a central role in health and disease. Immunol. Cell Biol. 78 (2), 91–102. doi: 10.1046/j.1440-1711.2000.00888.x 10762408 PMC7159383

[B40] Swenson D. L. Warfield K. L. Kuehl K. Larsen T. Hevey M. C. Schmaljohn A. . (2004). Generation of marburg virus-like particles by Co-expression of glycoprotein and matrix protein. FEMS Immunol. Med. Microbiol. 40 (1), 27–31. doi: 10.1016/s0928-8244(03)00273-6 14734183

[B41] Tseng C.-T. K. Perrone L. A. Zhu H. Makino S. Peters C. J. (2005). Severe acute respiratory syndrome and the innate immune responses: Modulation of effector cell function without productive infection. J. Immunol. SWJTU A+SCIIF 5 174 (12), 7977–7985. doi: 10.4049/jimmunol.174.12.7977 15944304

[B42] Wang C. Zheng X. Gai W. Zhao Y. Wang H. Wang H. . (2017). Mers-cov virus-like particles produced in insect cells induce specific humoural and cellular imminity in rhesus macaques. Oncotarget 8 (8), 12686–12694. doi: 10.18632/oncotarget.8475 27050368 PMC5355045

[B43] Xiong Y. Liu Y. Cao L. Wang D. Guo M. Jiang A. . (2020). Transcriptomic characteristics of bronchoalveolar lavage fluid and peripheral blood mononuclear cells in covid-19 patients. Emerging Microbes Infect. 9 (1), 761–770. doi: 10.1080/22221751.2020.1747363 PMC7170362 32228226

[B44] Yilla M. Harcourt B. H. Hickman C. J. McGrew M. Tamin A. Goldsmith C. S. . (2005). Sars-coronavirus replication in human peripheral Monocytes/Macrophages. Virus Res. 107 (1), 93–101. doi: 10.1016/j.virusres.2004.09.004p 15567038 PMC7114182

[B45] Yoshikawa T. Hill T. Li K. Peters C. J. Tseng C.-T. K. (2009). Severe acute respiratory syndrome (Sars) coronavirus-induced lung epithelial cytokines exacerbate sars pathogenesis by modulating intrinsic functions of monocyte-derived macrophages and dendritic cells. J. Virol. 83 (7), 3039–3048. doi: 10.1128/jvi.01792-08 19004938 PMC2655569

[B46] Young A. (2022). T Cells in sars-Cov-2 infection and vaccination. Ther. Adv. Vaccines Immunotherapy 10, 1–20. doi: 10.1177/25151355221115011 PMC9425900 36051003

[B47] Zepeda-Cervantes J. Ramírez-Jarquín J. O. Vaca L. (2020). Interaction between virus-like particles (Vlps) and pattern recognition receptors (Prrs) from dendritic cells (Dcs): Toward better engineering of vlps. Front. Immunol. 11. doi: 10.3389/fimmu.2020.01100 PMC7297083 32582186

[B48] Zhu N. Zhang D. Wang W. Li X. Yang B. Song J. . (2020). A novel coronavirus from patients with pneumonia in China, 2019. N Engl. J. Med. 382 (8), 727–733. doi: 10.1056/NEJMoa2001017 31978945 PMC7092803

